# Role of GTPase-Dependent Mitochondrial Dynamins in Heart Diseases

**DOI:** 10.3389/fcvm.2021.720085

**Published:** 2021-09-30

**Authors:** Jiangen Liu, Xianjing Song, Youyou Yan, Bin Liu

**Affiliations:** Department of Cardiology, The Second Hospital of Jilin University, Changchun, China

**Keywords:** heart disease, DRP1, mfn1, MFN2, OPA1

## Abstract

Heart function maintenance requires a large amount of energy, which is supplied by the mitochondria. In addition to providing energy to cardiomyocytes, mitochondria also play an important role in maintaining cell function and homeostasis. Although adult cardiomyocyte mitochondria appear as independent, low-static organelles, morphological changes have been observed in cardiomyocyte mitochondria under stress or pathological conditions. Indeed, cardiac mitochondrial fission and fusion are involved in the occurrence and development of heart diseases. As mitochondrial fission and fusion are primarily regulated by mitochondrial dynamins in a GTPase-dependent manner, GTPase-dependent mitochondrial fusion (MFN1, MFN2, and OPA1) and fission (DRP1) proteins, which are abundant in the adult heart, can also be regulated in heart diseases. In fact, these dynamic proteins have been shown to play important roles in specific diseases, including ischemia-reperfusion injury, heart failure, and metabolic cardiomyopathy. This article reviews the role of GTPase-dependent mitochondrial fusion and fission protein-mediated mitochondrial dynamics in the occurrence and development of heart diseases.

## Introduction

Over the course of a human lifetime, the heart beats ~2.5 billion times. The enormous amount of energy required for this cyclic beating process is provided by the mitochondria of myocardial cells. Mitochondria are membrane-bound organelles found in the cytoplasm of almost all eukaryotic cells (cells with clearly defined nuclei), the primary function of which is to generate large quantities of energy in the form of adenosine triphosphate (ATP). Aside from their role in energy production, mitochondria also have a vital role in maintaining cell homeostasis.

Mitochondria are organelles whose morphology and function are interconnected. Through the dynamic balance of fission and fusion, the normal morphology of mitochondria is regulated to meet the energy and metabolic needs of the cell. These dynamic changes in morphology maintain the integrity and distribution of mitochondria, thereby allowing their function to adapt to changing physiological needs (1, 2). Mitochondria are highly abundant and widely distributed throughout the cytoplasm of cardiomyocytes. In fact, more than 40% of the cytoplasmic space of adult cardiac cardiomyocytes is occupied by densely packed mitochondria (3), which are primarily located between the sarcomeres of cardiomyocytes, around the nucleus, and under the serosa (3), allowing them to continuously provide energy for myocardial contraction. Therefore, the morphology of mitochondria is essential to maintain the health of cardiomyocytes. Cardiac mitochondria can be divided into two types based on their location, namely, subserosa mitochondria and interfiber mitochondria. The cristae morphology of these two mitochondrion types differs; that is, the subserosa mitochondria are flaky, whereas interfiber mitochondria are primarily tubular. However, cristae morphology can change in response to the pathophysiological state of heart disease, suggesting that mitochondrial morphology also has an important role in cardiomyocyte homeostasis (4).

**Figure 1:**
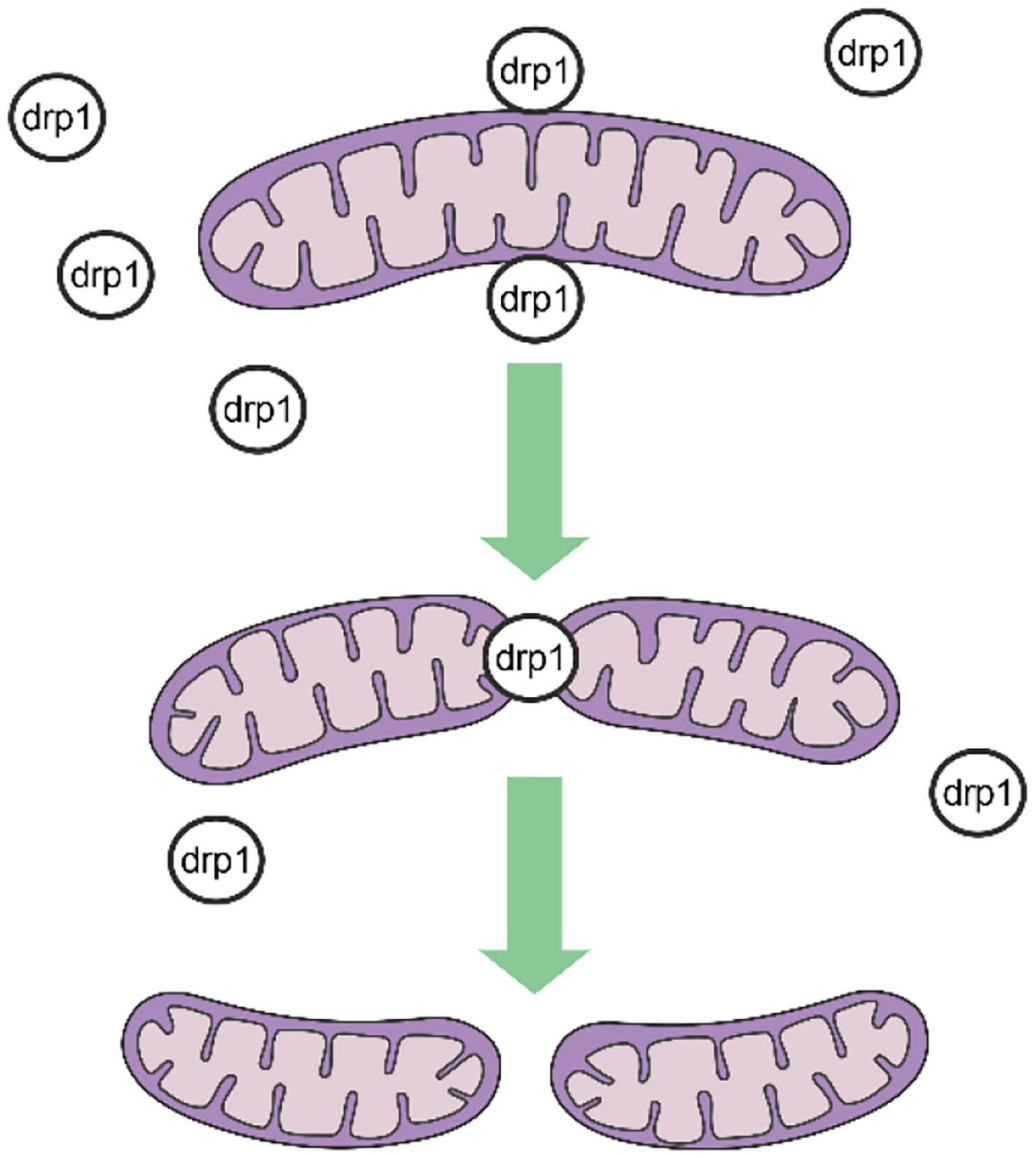
Procedures of mitochondrial fission.

**Figure 2:**
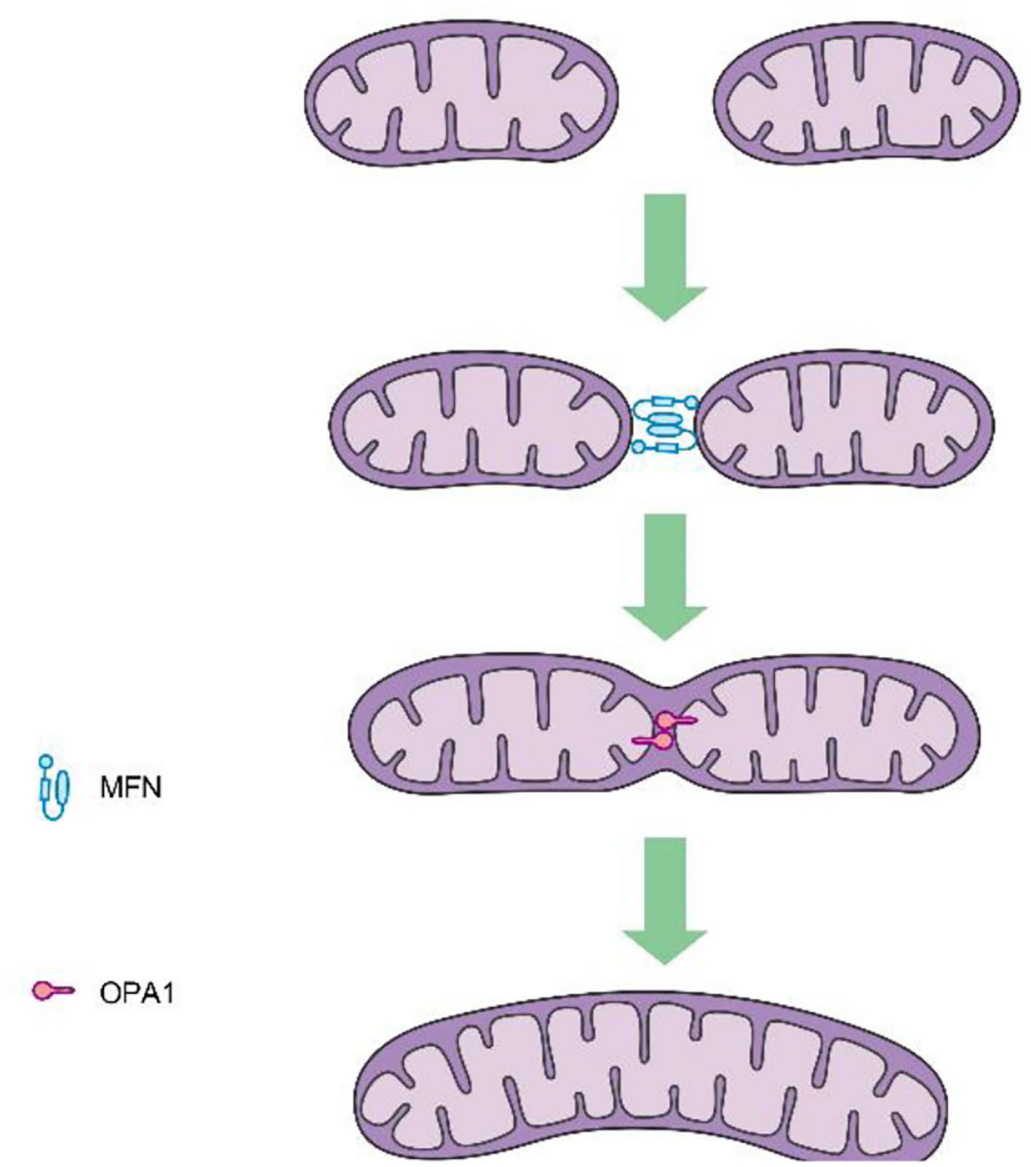
Procedures of mitochondrial fusion.

Mitochondrial fission is mediated by dynamin-related protein 1 (DRP1), a GTPase-dependent enzyme that is recruited to the outer mitochondrial membrane through a series of receptor proteins (MFF, FIS1, MID49, and MID50) (5). Together, DRP1 and FIS1 mediate fission and participate in apoptosis activation, necrosis, and autophagy (6), after which DRP1 divides the mitochondria into two in a GTP-dependent manner (See in Figure 1). In mammals, mitochondrial fusion is generally regulated by three GTPase-dependent proteins in the dynamin superfamily, namely, mitofusin-1 (MFN1), mitofusin-2 (MFN2), and optic atrophy 1 (OPA1), all of which participate in the processes of energy metabolism, mitochondrial permeability transition, and calcium homeostasis (6, 7). Generally, mitochondrial fusion requires three steps. First, MFN1 or MFN2 reversibly approach the outer mitochondrial membranes. Second, the outer membranes of the two mitochondria fuse *via* MFN1 and MFN2 under GTPase mediation. Third, the inner mitochondrial membranes fuse *via* OPA1 in a GTPase-dependent manner (Figure 2).

In most organs, mitochondrial dynamics are highly active, with mitochondria continually undergoing fusion, fission, transportation, and degradation. These dynamic processes are necessary to maintain the normal shape, health, and number of mitochondria, thus, maintaining cellular physiological functioning. Yet, the description of mitochondria in a hyperdynamic state may not be suitable to adult cardiomyocytes, where the mitochondria appear as independent, lowly dynamic organelles. Meanwhile, under stress or pathological conditions, morphological changes have been observed in myocardial mitochondria. Moreover, the delicate balance between fission and fusion is critical to ensure a steady energy supply and cardiomyocyte hemostasis (8). To date, many studies have confirmed that the fusion and fission dynamins of cardiac mitochondria, in particular the GTPase dependent dynamins (DRP1, MFN1, MFN2, OPA1) are associated with the occurrence and development of various heart diseases. As such, this article provides a review of current literature related to the effects of these specific GTPase-dependent dynamins that mediate mitochondrial fusion and fission in myocardial tissue.

## Role of GTPase-Dependent DRP1 in Heart Diseases

### DRP1 Structure and Modification

Along with mitochondrial fusion, fission is essential to maintain normal cell and tissue physiological functions (9, 10). Mitochondrial fission is mediated by the GTPase-dependent dynamin DRP1, which is composed of four fragments: the N-terminal GTPase active fragment, middle fragment, variable fragment, and C-terminal GTPase effector fragment. Before Drp1 participates in the fission of mitochondria, a series of proteins are needed to transport it to the outer mitochondrial membrane. These proteins are called Drp1 Adaptors. The adaptors include fission 1 (Fis1), mitochondrial fission factor (Mff) and mitochondrial dynamics proteins of 49 and 51 kDa (MiD49, MiD51) (5). And MiD49 and MiD51 can act independently of Mff and Fis1 in Drp1 recruitment and suggest that they provide specificity to the division of mitochondria (11). A number of reports have shown that Fis1 is not required for Drp1 recruitment, but Mff is an essential factor for mitochondrial recruitment of Drp1 during mitochondrial fission in mammalian cells (12).

**Figure 3:**
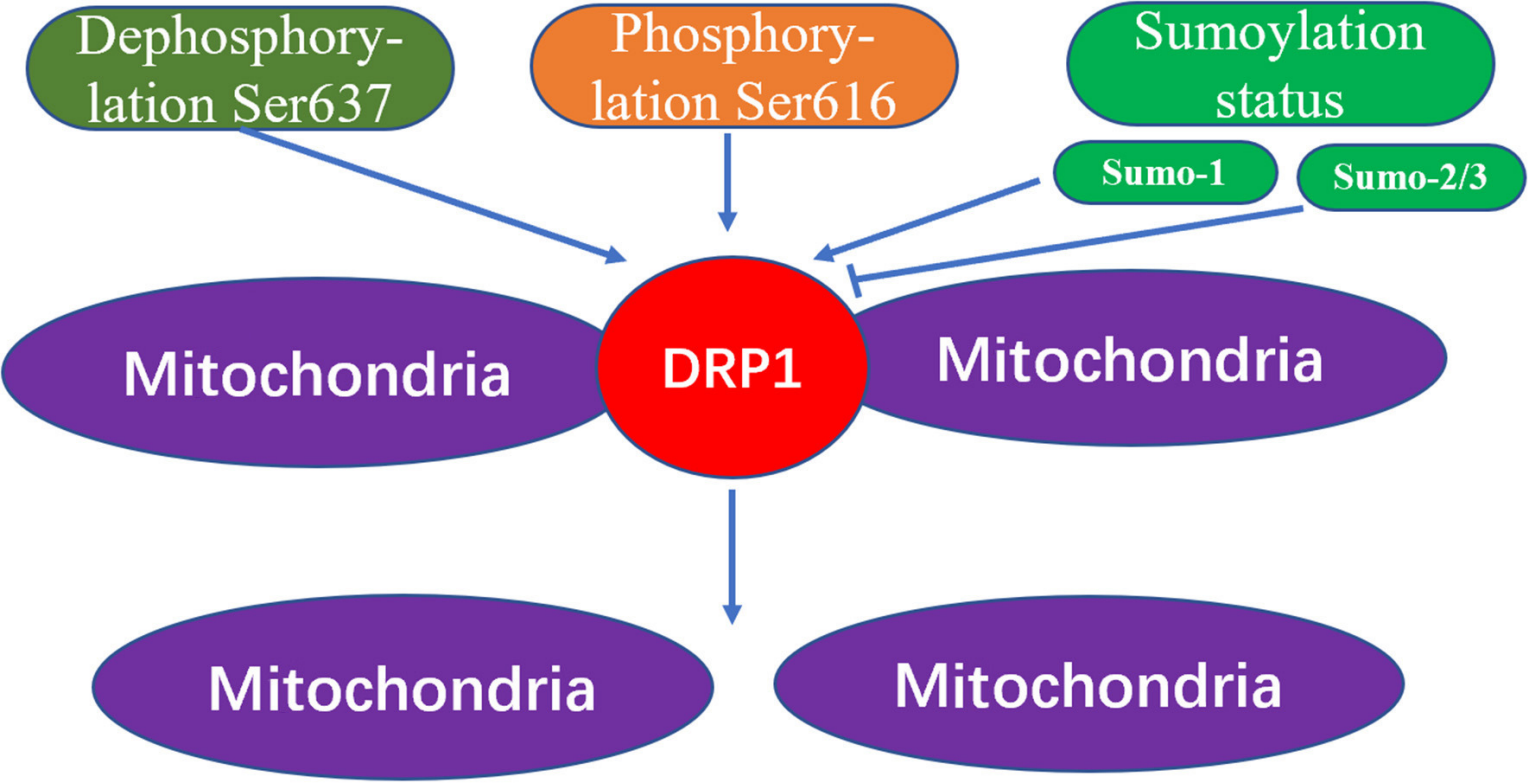
The mechanism of mitochondrial fission caused by Drp1 modification. The increased dephosphorylation of Ser637 on the drp1 protein or the increased phosphorylation of Ser616, and the change of the sumoylation state of DRP1 will effect the activity of DRP1, thereby increasing the fission of mitochondria.

Through the action of various kinases and phosphatases, the C-terminal GTPase effector fragment is reversibly phosphorylated to achieve DRP1 aggregation on the surface of mitochondria (13, 14). This DRP1 aggregation allows for mitochondria reshaping in response to intracellular or extracellular signals. Phosphorylation of the commonly confirmed DRP1 sites, Ser616 and Ser637, represents important regulatory mechanisms following DRP1 translation, whereby Ser616 phosphorylation induces DRP1 activity, while Ser637 phosphorylation reduces DRP1 activity (15). For example, during cell mitosis, CDK1 enhances mitochondrial division through Ser616 phosphorylation (16). Meanwhile, phosphorylation of Ser637 on the DRP1 C-terminal GTPase effector fragment by cAMP-dependent protein kinase, reduces the GTPase activity of DRP1 and inhibits mitochondrial fission (17). In contrast, dephosphorylation of Ser637 mediated by calcium-dependent phosphatase and calcineurin increases DRP1 recruitment from the cytoplasm to the mitochondria (18). Additionally, the sumoylation status of DRP1 can also affect its intracellular localization; that is, when DRP1 is modified by SUMO-1, localization of DRP1 to mitochondria increases (19, 20), while DRP1 modification by SUMO-2/3 inhibits its localization to mitochondria (21) (Figure 3).

### The Role of DRP1 in Embryogenesis and Heart Development

Endogenous DRP1 plays an important role in mitochondrial quality control and cardiomyocyte survival. For example, *Drp1* knock out in mice leads to embryonic lethality, where the embryos generally die within 9.5–11.5 embryonic days, a phenotype accompanied by elongated mitochondria, decreased cell differentiation, and decreased developmental regulatory apoptosis (22). Moreover, targeted knock out of *Drp1* in murine heart tissue after birth caused dilated cardiomyopathy and death. Meanwhile, the cardiomyocyte mitochondria exhibited internally aggregated ubiquitin proteins accompanied by weakened cell respiration (23–25). The effects associated with DRP1 were also demonstrated with a mutation scan of N-ethyl-N-nitrosourea, in which mice with *Drp1* mutations developed dilated cardiomyopathy related to reduced levels of respiratory complexes and loss of ATP (26). Additionally, three studies that specifically knocked out *Drp1* in murine cardiac tissues reported that lack of DRP1 led to enlarged mitochondria, while DRP1 loss was fatal during the perinatal period or in the mature heart. Moreover, mitochondrial quality and abundance were found to be related to mitochondrial autophagy (24, 27, 28). The specific deletion of myocardial *Drp1* not only increases the mitochondrial size, but also causes their abundance to progressively decrease (23).

In mice with muscle-specific *Drp1* knockout, all pups died between 7 and 10 days after delivery, however, the death was not accompanied by neonatal defects or weight changes. Meanwhile, the hearts of *Drp1* knockout mice were enlarged 7 days after delivery, accompanied by a thinning of the heart walls, however, the heart weight remained normal. Ultrasonography revealed clear cardiac dysfunction, including an increase in the end-systolic diameter (0.85 mm in the control compared to 1.64 mm in the *Drp1* knockout) and a decrease in the shortening rate of the left ventricular short axis (46.5% in the control compared to 21.4% in the *Drp1* knockout). Furthermore, heart-specific knockout of *Drp1 via* gene recombination after birth caused cardiomyopathy and death in young mice (28). Collectively, these results indicate that DRP1 is necessary for the maintenance of neonatal heart function.

DRP1 clearly plays an important role in embryogenesis and heart development in newborn mice. During these developmental stages, *Drp1* inhibition or knock out causes enlargement and excessive fusion of mitochondria, both of which are lethal to mice. Hence, DRP1 has important roles in different stages of ontogeny. Under normal physiological conditions, inhibiting or silencing *Drp1* reduces mitochondrial fission, increases fusion, and causes loss of heart function and even death. Under pathological conditions, the DRP1 expression becomes increased; its subsequent excessive phosphorylation or acetylation cause apoptosis and autophagy resulting in excessive mitochondrial fission and reduced fusion, thus further inducing cardiac dysfunction.

### The Role of DRP1 in Pathological Cardiac Conditions

One study found that within 60 min of ischemia reperfusion, DRP1 expression increased with excessive mitochondrial fission following localization of DRP1 and BAX (apoptosis-related protein) to the mitochondria (29). During this process, MFN1 and MFN2 activity was inhibited, which may have led to induced activation of the mitochondrial permeability transition pore (MPTP) (30), causing subsequent mitochondrial fragmentation and permeation of the outer membrane (31, 32). Increased mitochondrial outer membrane permeability or opening of the MPTP leads to cytochrome C release, thus, further activating the caspase apoptosis pathway (33, 34). In the case of ischemia-reperfusion injury, inhibition of DRP1 activity elicits a protective effect on the heart. Thus, in different stages of the life cycle and development, as well as during physiological and pathological states, the degree of change in DRP1 activity differs, leading to different effects on cardiomyocytes and the heart.

As detailed above, the excessive mitochondrial fission observed within 60 min of reperfusion after transient ischemia, led to mitochondrial dysfunction and a decrease in myocardial contractility. However, P110, an inhibitor of DRP1, can reverse the decrease in myocardial contractility, thereby improving heart function (35). During reperfusion, dephosphorylation of Ser637 on DRP1 induces the transfer of DRP1 to the mitochondrial membrane and enhances mitochondrial fission (36). This dephosphorylation event in ischemia-reperfusion injury can be mediated by calcineurin (36, 37). Thus, because FK506 is an inhibitor of calcineurin, it can inhibit Ser637 dephosphorylation and ultimately preserve cardiac function during ischemia-reperfusion injury (36). In addition, AKT (protein kinase B) can activate DRP1 (38). For instance, in diabetes, the level of SIRT1 (sirtuin 1) in cardiomyocytes decreases, causing increased phosphorylation of Ser473 on AKT, leading to increased dephosphorylation of Ser637 on DRP1, thereby enhancing DRP1 activity, which subsequently increases mitochondrial fission and aggravates cardiac ischemia-reperfusion injury (39). Meanwhile, mammalian STE20-like kinase 1 (MST1) is upregulated after myocardial infarction and increases mitochondrial fission through the JNK-DRP1 (DJ-1) cell signal transduction pathway, leading to cardiomyocyte fibrosis and aggravating myocardial damage (40). Moreover, DJ-1 activation can alter the sumoylation state of DRP1, thereby inhibiting mitochondrial division, and playing a protective role in ischemia-reperfusion injury. A potential mechanism underlying this process may involve the interaction of DJ-1 with SENP5 to enhance DRP1 sumoylation mediated by SUMO-2/3, which is modified to inhibit mitochondrial division (41).

By inhibiting the DRP1 expression mitochondrial fission is also inhibited, which can alleviate cardiac ischemia-reperfusion injury. At the same time, increased mitochondrial fusion occurs in these DRP1-inhibited cardiomyocytes (36, 42, 43). These studies assessed the effect of mitochondrial fission in cultured cardiomyocytes by RNA interference and *via* expression of the main negative form of DRP1. That is, adenovirus-transfected cells overexpress the negative regulator of DRP1, K38A *in vivo* and *in vitro*, which subsequently protects myocardial cells from the damage associated with ischemia-reperfusion (43). In cardiac ischemia, endogenous levels of the microRNA miR-499 are downregulated, while calcineurin is activated, and Ser637 on DRP1 is dephosphorylated, thus, further increasing mitochondrial fragmentation, and in turn leading to increased apoptosis of cardiomyocytes. Thus, miR-499 overexpression inhibits calcineurin-mediated DRP1 dephosphorylation, thereby reducing cardiomyocyte apoptosis (44). miR-499 can also inhibit DRP1 through the cAMP-PKA cell signal transduction pathway, thereby prolonging the mitochondrial lifespan (17). SIRT3 can further reduce the phosphorylation level of DRP1 by normalizing AMPK cell signal transduction pathways, thus, inhibiting mitochondrial fission and ultimately reducing myocardial damage following myocardial infarction (45). Similarly, treatment of mice with the DRP1 inhibitor Mdivi-1 leads to decreased mitochondrial fission, thus imparting a protective effect against hypoxia and ischemia reperfusion (36, 42). Moreover, *in vitro*, a simulated ischemia-reperfusion model with cultured HL-1 cells pretreated with Mdivi-1 improved cell survival and delayed the opening of MPTP (42). In Mdivi-1 pretreated mice, reperfusion in the occluded artery and the area of myocardial infarction were found to decrease (42). In fact, emergency inhibition of mitochondrial division after acute myocardial infarction can improve long-term cardiac function (35, 36). Meanwhile, in a mouse cardiac arrest model, inhibition of myocardial mitochondria fission improves the survival rate of mice (46). In pressure load-induced heart failure, Mdivi-1 can also improve left ventricular function and reduce myocardial fibrosis (47). Furthermore, as an inhibitor of DRP1, P110 can inhibit the upregulation of the autophagy marker LC3-II whether it is applied before or after cardiac reperfusion, however, it also inhibits the increase in phosphorylation of cleaved caspase 3 and JNK when applied after reperfusion, both of which are associated with cell apoptosis and death (35, 48, 49). Indeed, treatment of the heart with Mdivi-1 and P110 elicited a protective effect against ischemia-reperfusion injury (35, 42, 50), as DRP1 inhibitors not only maintain mitochondrial morphology but also reduce calcium ions in the cytoplasm, inhibit the opening of MPTP, and inhibit cardiomyocyte apoptosis, thereby reducing myocardial damage. In fact, a recent study showed that injecting Mdivi-1 prior to myocardial infarction in ischemic mice can increase the length of mitochondria in myocardial cells (51). However, other studies that injected Mdivi-1 into the coronary arteries of larger animals, including pigs, as models for cardiac reperfusion after acute myocardial infarction, reported no significant changes in mitochondrial morphology between the blank control and experimental animals. Moreover, a protective cardiac effect was not detected, which may be related to the dose of Mdivi-1, timing of application, method of administration, etc. (52). Further studies are required to resolve these discrepancies in *in vivo* findings; hence, much work is still required before inhibitors of DRP1 can be recommended for the clinical treatment of heart diseases.

Diabetic cardiomyopathy refers to the abnormal structure and function of the heart caused by diabetes after excluding dangerous factors, including hypertension, coronary heart disease, and valvular disease, which may cause cardiac insufficiency. Mitochondrial dysfunction plays an important role in diabetic cardiomyopathy (53). Within minutes, acute hyperglycemia conditions can induce formation of shortened mitochondria mediated by the fission protein DRP1 (54). The acute response depends on the post-translational modification mechanism, which may involve glucose-induced intracellular calcium transients and ERK1/2 activation, causing phosphorylation of Ser616 on DRP1 (55). Specifically, PKCδ, CDK1, and CDK5 phosphorylate Ser616 to increase the fission activity of DLP1 (16, 56, 57). Melatonin regulates the expression of DRP1 through the SIRT1-PGC1α cell signal transduction pathway to reduce mitochondrial fission and ultimately alleviate diabetes-induced cardiac insufficiency (58). The rapid formation of small mitochondria under acute metabolic stress leads to an increase in mitochondrial reactive oxygen species (ROS). When the blood sugar continues to rise, the pathological fragmentation of mitochondria is related to the chronic increase in ROS levels, apoptosis, and necrosis (54, 59).

In the hearts of rats with chronic hypertension and hypertrophy, the level of cardiac DRP1 is reduced (60); however, myocardial DRP1 levels are elevated in the hypertrophic heart induced by neurohormone-norepinephrine (61). Studies have confirmed that mitochondria play an important role in cardiac hypertrophy (26, 62). For example, disorganized and fragmented mitochondria have been shown to cause cardiac hypertrophy (61, 63). Meanwhile, *in vitro*, within the NE (Norepinephrine)-induced cardiomyocyte hypertrophy model, an increase in accumulated DRP1 on the surface of mitochondria, as well as an increase in mitochondrial fragmentation, and a decrease in mitochondrial capacity are observed. However, if adenovirus-transfected cells are used to inhibit the expression of DRP1, mitochondrial fission is reduced, alleviating cardiomyocyte hypertrophy (61). Angiotensin-(1–9) can regulated mitochondria morphology through the AT2R/miR-129-3p/PKA cell signal transduction pathway, thereby inhibiting mitochondria fission and further reducing the occurrence of cardiomyocyte hypertrophy (64). Moreover, leptin can induce cardiac hypertrophy and activate calcineurin (65). The dephosphorylation of Ser637 on DRP1 mediated by calcineurin increases the recruitment of DRP1 from the cytoplasm to the mitochondria (18). It is, therefore, thought that leptin-induced cardiomyocyte hypertrophy is related to enhanced mitochondrial fission. In the cardiac hypertrophy cell model, fission of mitochondria has also been shown to participate in the calcium-dependent calcineurin cell signal transduction pathway (61). Mitochondrial fission is also crucial for the maintenance of heart function. Targeted *Drp1* knock out in the adult mouse heart induced development of cardiomyopathy after 6–13 weeks (27, 28). Moreover, mitochondrial fission was significantly inhibited; however, this failed to inhibit the occurrence of heart failure. Meanwhile, in a murine model of pressure overload, Mdivi-1 reduced myocardial hypertrophy and fibrosis without affecting blood pressure (66). Similarly, in an aortic constriction model in which *Drp1* was specifically knocked out of cardiac tissue cells *via* persistent heterozygous, excessive hypertrophy of cardiomyocytes and decreased heart function were observed. At the same time, it was confirmed that DRP1-dependent mitochondrial autophagy plays a role in resisting pressure load. With this important role, DRP1-dependent mitochondrial autophagy can ensure normal abundance of mitochondria and ultimately, contribute to amelioration of decreased heart function (67). Moreover, during heart failure, IGF-IIR can enhance the phosphorylation of Ser616 of DRP1 through extracellular signal-regulated kinase (ERK), thereby enhancing mitochondrial fission and causing mitochondrial dysfunction (68).

**Figure 4:**
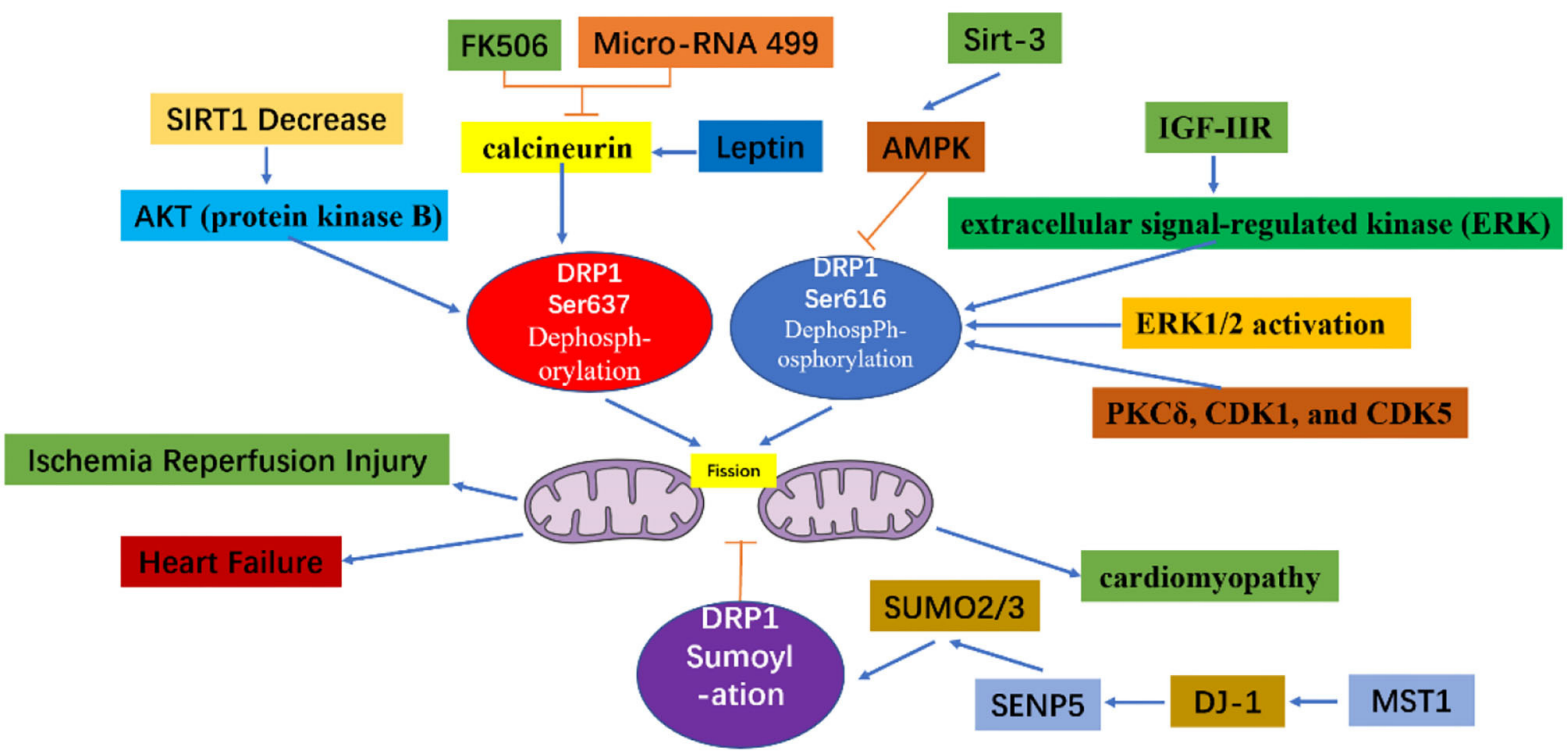
Cell signal transduction pathway acting on DRP1 and its role in heart disease.

To make a summary of the whole section, DRP1 plays an important role in both the embryonic development period and the development of the newborn heart. DRP1 is elevated in ischemia-reperfusion injury causing excessive mitochondrial fission, thereby, further aggravating ischemia-reperfusion injury. DRP1 also plays an important role in the occurrence and development of heart failure, hence, inhibiting DRP1 can reduce ischemia-reperfusion injury and heart failure (Table 1). Specifically, DRP1 can affect mitochondrial morphology and function through phosphorylation, acetylation, sumoylation, and other modifications (Figure 4). Thus, DRP1 affects the occurrence and development of heart disease, which may be related to the imbalance of mitochondrial fission and fusion; however, further research is required to understand the specific mechanisms involved.

**Table 1:** Drp1 the role of Drp1 in heart development and heart disease.

**Diseases**	**GTP-ase dependent dynamin state**	**Methods**	**Effect on the heart**	**References**
Embryogenesis and heart development		Global Knock out in mice	Embryonic lethality	(22)
		Heart specifically knocked out	Dilated cardiomyopathy and death	(28)
Ischemia-Reperfusion	DRP1 activation Increase	Mdivi-1,P110 Drp1 inhibitor	Reverse the decrease in myocardial contractility, thereby improving heart function	(35, 36, 42, 47, 50, 51)
		Inhibit Ser637 dephosphorylation	Preserve cardiac function	(36, 37)
		DRP1 sumoylation mediated by SUMO-2/3	Protect the heart from ischemia-reperfusion injury	(41)
Cardiac hypertrophy	DRP1 activation Increase	Inhibit the expression of DRP1	Alleviate cardiomyocyte hypertrophy	(61, 64)
		Mdivi-1 Drp1 inhibitor	Reduced myocardial hypertrophy and fibrosis	(66)

## Role of GTPase-Dependent Fusion Proteins in Heart Diseases

Mitochondrial fusion is regulated by GTPase-dependent proteins, including MFN1, MFN2, and OPA1. Mitochondrial fusion plays an important role in the maintenance of mitochondrial DNA homeostasis. In cultured cells and skeletal muscle cells lacking mitochondrial fusion factors, mtDNA decreased and mtDNA accumulation mutation increased (69).

### Role of GTPase-Dependent Mitofusins in Heart Diseases

#### Role of Mitofusins (MFN1 and MFN2) in Heart Diseases

Mammalian mitochondrial fusion protein has been identified as a human homolog of the Drosophila fuzzy onion protein (Fzo) (70). MFN1 is composed of 741 amino acids, whereas MFN2 is composed of 757 amino acids. Both are transmembrane GTPases located on the outer mitochondrial membrane, with functional domains that share 63% homology. The conserved domain of mitofusins consists of an amino-terminal GTP binding domain, a coiled-coil domain (heptapeptide repeat HR1), a carboxy-terminal with a two-part transmembrane domain and a second coiled-coil domain (heptapeptide repeat sequence HR2). Both the GTPase and coiled-coil domains are exposed to the cytoplasm. In addition, MFN2 possesses an N-terminal Ras binding domain that does not exist in MFN1, implying that MFN2 has unique functions that are not shared with MFN1 (71). Although both MFN1 and MFN2 support mitochondrial fusion, MFN2 also mediates the coupling of the endoplasmic reticulum and mitochondria (72). Therefore, MFN2 plays a critical role in maintaining intercellular calcium regulation, which is of great significance to the excitation-contraction-metabolic coupling of cardiomyocytes. Unsurprisingly, MFN2 is highly abundant in the heart compared to other tissues (73).

Cardiac-specific *Mfn1* and *Mfn2* knockout mice exhibit death due to heart failure, respiratory dysfunction, and mitochondrial fragmentation and swelling. In dual *Mfn1* and *Mfn2* cardiac knockout mice, no births were observed with embryos dying on embryonic days 9.5 and 10.5. However, the expression of *Mfn1* or *Mfn2* alone is sufficient for murine survival, thus only the simultaneous absence of both is incompatible with life (74). In single *Mfn1* or *Mfn2* knockout mice, mitochondrial outer membrane fusion and aggregation proceed normally, while double knockout result in loss of fusion induced by mitofusins (75). In 8-week-old mice, when *Mfn1* and *Mfn2* were specifically and simultaneously knocked out in cardiac tissue, the cardiomyocytes showed mitochondrial fragmentation and respiratory dysfunction, rapidly developing into lethal dilated cardiomyopathy (76). Additionally, MFN1 levels have been found to decrease in myocardial hypertrophy (77), while MFN2 levels increase under oxidative stress (78), however, MFN2 levels in the late stage after myocardial infarction and diabetis late stageare significantly reduced (79, 80). A recent study showed that microRNA-20b can downregulate *Mfn2* and promote cytoplasmic Ca^2+^ overload, thus, weakening the buffering capacity of mitochondria and increasing cardiac hypertrophy (81).

In mice with *Mfn1* only knockout, cardiac function, mitochondrial respiratory function, and respiratory function are maintained; however, an increase in spherical mitochondria can still be observed (82, 83). In contrast, cardiac *Mfn2* ablation causes early-dilated cardiomyopathy in mice. Indeed, MFN1 loss is better tolerated than MFN2 loss in cardiomyocytes (30, 84), which may account for why single *Mfn1* knockout elicit minimal effects on heart function. These data imply that when either MFN1 or MFN2 are missing, the other protein may compensate for the lost function, however, this requires further verification.

In ischemia-reperfusion injury, DRP1 and BAX are transferred to mitochondria (29). In this process, the activities of MFN1 and MFN2 are inhibited, resulting in mitochondrial fragmentation and outer membrane permeability (31, 32). MFN inhibition can induce the activation of MTPT (30). When either mitochondrial outer membrane permeability is increased or the mitochondrial permeability transition pore opens, cytochrome C is released, further activating the caspase cell apoptosis pathway (33, 34). In the simulated ischemia-reperfusion injury model, the overexpression of MFN1 or MFN2 in the HL-1 cardiomyocyte cell line reduces cell death (42). Meanwhile, following siRNA silencing of *Mfn2* expression in a rat neonatal cardiomyocyte ischemia-reperfusion injury model, the cell survival rate was significantly decreased (77). Acute knockout of *Mfn1* and *Mfn2* in the heart showed a short-term protective effect against acute myocardial infarction, related to the decreased contact between mitochondria and endoplasmic reticulum, thereby reducing the calcium overload in the mitochondria (76). In response to ischemia-reperfusion injury, mitochondria transport calcium ions from the endoplasmic reticulum to the mitochondria through a calcium ion unidirectional transport pump. Upregulation of calcium ions in the mitochondria can induce the opening of the MPTP, which is a determinant of cardiomyocyte death (85). In ischemia-reperfusion injury, inhibiting calcium overload in mitochondria can reduce myocardial cell death and the myocardial infarction area (86–88). Moreover, MFN2 reportedly has an important role in the connection between mitochondria and the endoplasmic reticulum, ensuring sufficient calcium transfer for the production of bioenergy (72). After its activation, βII protein kinase C can phosphorylate Ser86 on MFN1, effectively inhibiting its GTPase activity and leading to fragmentation and dysfunctional mitochondrial accumulation, which in turn causes deterioration of heart function (89).

#### Role of OPA1 in Heart Diseases

OPA1 is a GTPase-dependent protein that mediates the fusion of mitochondria, while organizing the morphological regeneration of mitochondrial cristae, and resisting apoptosis for physiological needs (7, 90, 91). Evidencing the important role of OPA1 in the maintenance of mitochondrial morphology, knocking out *Opa1* during embryogenesis is fatal (92). In fact, *Opa1* or *Mfn2* knockout, *via* gene trapping, prevents embryonic stem cells (ESCs) from differentiating into cardiomyocytes (93). Mitochondrial fusion disrupts and increases the entry of capacitive calcium, thereby enhancing Notch1 signaling through calcium-induced calcineurin activation. Meanwhile, inhibition of calcium and calcineurin signals is sufficient to reverse the non-differentiated phenotype of gene-trapped ESCs without affecting mitochondrial morphology. This places mitochondrial dynamics upstream of Notch signaling ESC differentiation in cardiomyocytes, thereby supporting the regulation of mitochondrial dynamics in heart development.

Wai et al. (94) observed morphological changes in the mitochondria of hearts from *Yme1l* knockout mice, finding that the smaller mitochondria aggregated, while their cristae structure was normal. This indicates that mitochondrial dynamics were damaged due to YME1L loss. Furthermore, in cardiomyocytes lacking YME1L, the D form of s-OPA1 disappears, while the C and E forms of s-OPA1 aggregate as a result of OMA1 activation. Fragmentation of the mitochondrial network was also found in fibroblasts with *Yme1l* knocked out *in vitro*, and cell damage caused by s-OPA1 aggravation was also observed. The absence of YME1L in cardiomyocytes activates OMA1, which further increases the processing level of OPA1, increases the fragmentation of mitochondria, and ultimately leads to dilated cardiomyopathy and heart failure. In ischemia-induced heart failure, OPA1 expression decreases, indicating that OPA1 plays an important role in ischemic cardiomyopathy (95). In *Drosophila* and mice, inhibiting *Opa1* expression may cause abnormal mitochondrial morphology and cardiac hypertrophy (96, 97). Therefore, the inhibition of mitochondrial fusion-related proteins can increase mitochondrial fragmentation, which further affects the arrangement of cardiomyocytes and sarcoplasmic reticulum, thus, impacting cardiac function.

The change in OPA1 form represents a key step in mitochondrial cooperative fusion and fission regulation (98, 99). The balance between long and short forms of OPA1 maintains the normal shape of mitochondria: fusion depends on long OPA1, while short OPA1 is related to mitochondrial fission (100, 101). Cell pressure, mitochondrial dysfunction, or genetic intervention (such as *Yme1l* deletion) can activate OMA1, thereby increasing the conversion of long OPA1 to short OPA1, and increasing the fragmentation of mitochondria (100, 102–104). Thus, GTPase OPA1 located on the inner mitochondrial membrane is a key enzyme that regulates mitochondrial fission-fusion. Mitochondrial protease OMA1 and AAA protease (AAA proteases comprise a conserved family of membrane bound ATP-dependent proteases that ensures the quality control of mitochondrial inner-membrane proteins) YME1L can cleave OPA1 from its long to short form and, while short OPA1 does not play a role in mitochondrial fusion, the aggregation of short OPA1 may cause accelerated fission. In cultured mammalian cells, OMA1 is activated under pressure, which leads to increased mitochondrial fission (94, 105, 106). Sadhana et al. (107) confirmed that SIRT3 can regulate the enzymatic activity of OPA1 through lysine deacetylation. Mitochondrial respiration depends on the acetylation state of the C-terminal K926 and K931 lysine fragments in the GED region of the OPA1 protein. Cardiac stress causes hyperacetylation of OPA1, thereby reducing its GTPase activity, however, SIRT3 can directly bind to OPA1, thereby enhancing mitochondrial function and networking (107). Studies have found that OPA1 is acetylated in the states of cardiac hypertrophy and SIRT3 deficiency. Overexpression of SIRT3 can play a protective role in the process of adriamycin-mediated mitochondrial fragmentation and cell death. These mechanisms are all related to the acetylation status of OPA1. SIRT3 exerts its activity by directly binding to OPA1 on the inner mitochondrial membrane and modifying it by deacetylation. SIRT3 maintains or strengthens the mitochondrial respiratory complex by activating OPA1. SIRT3 can inhibit mitochondria-mediated apoptosis by mediating OPA1. Meanwhile, hyperacetylation reduces the GTPase activity of OPA1, indicating that the decreased activity of OPA1 may partially account for mitochondrial defects in hypertrophy of the heart.

Normal expression of OPA1 is necessary for the maintenance of mitochondrial autophagy. The expression of OPA1 is decreased in infarcted hearts *in vivo* or in cardiomyocytes cultured *in vitro*. Meanwhile, within cardiomyocytes, irisin can activate OPA1-induced mitochondrial autophagy (108). In myocardial ischemia-reperfusion injury, decreased expression of OPA1 leads to increased mitochondrial fragmentation, and thus mitochondrial dysfunction and increased mitochondrial apoptosis, further aggravating ischemia-reperfusion injury (109). The increase or overexpression of OPA1 maintains mitochondrial function and cardiac function during myocardial ischemia and reperfusion (110). In contrast, in H9C2 cells cultured *in vitro*, overexpression of OPA1 leads to mitochondrial lengthening with no detectable reduction in apoptosis under ischemic stimulation (95). OPA1 levels decrease in failing hearts and in late stages after myocardial infarction (80, 95), while increasing in hypertensive hypertrophic hearts and cardiomyocytes treated with insulin (60, 111). MFN1, MFN2, and OPA1 levels showed a consistent decrease in the genetically induced lipid-overloaded heart (112). The stability of OPA1 in cardiomyocytes is regulated by ERK, AMPK, and YAP cell signal transduction pathways (113).

Melatonin can activate OPA1-mediated mitochondrial autophagy and fusion in an AMPK-dependent manner, thereby reducing cardiac ischemia-reperfusion injury (110). Coenzyme Q10 can induce the AMPK-YAP-OPA1 cell signal transduction pathway, thus, improving mitochondrial function and reducing the degree of arteriosclerosis (114). Calenduloside E can reduce cardiac ischemia-reperfusion injury by regulating OPA1-related mitochondrial fusion mediated by AMPK activation (115). In the cardiomyocyte model of ischemia-reperfusion injury, increased expression of OPA1 can reduce the oxidative stress of cells through the Ca^+2^/calmodulin-dependent protein kinase II (CaMKII signaling) pathway (116).

**Figure 5:**
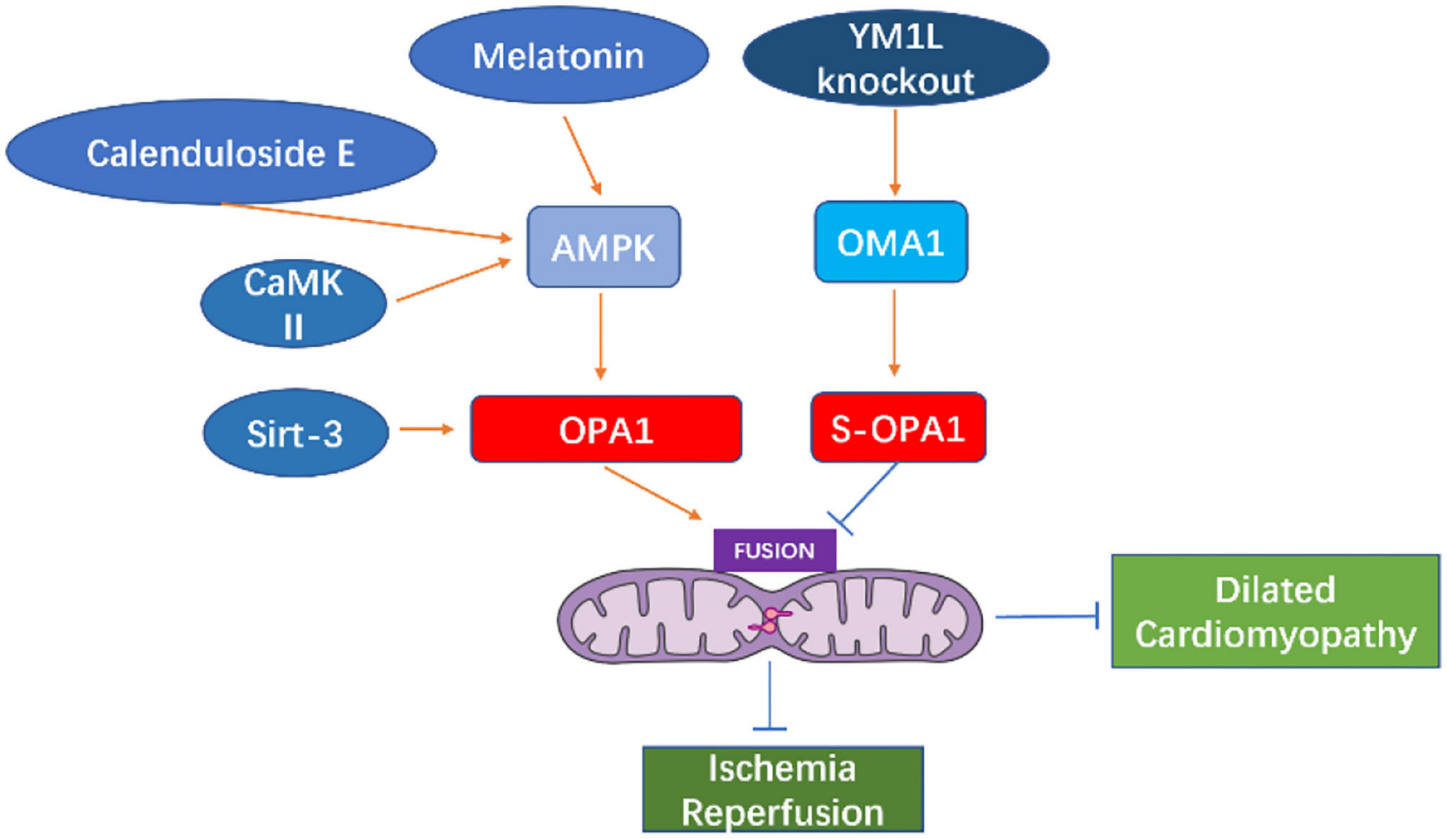
Cell signal transduction pathway acting on OPA1 and its role in heart disease.

Studies in murine diabetes models have shown that O-GlcNAc of OPA1 and DLP1 increase mitochondrial fragmentation (117, 118). O-GlcNAc modification of OPA1 can inhibit its function, leading to fragmentation of mitochondria, reduction of mitochondrial membrane potential, and reduction of complex IV activity (118). It is reported that enhanced O-GlcNAc modification of diabetic cardiomyopathy (DCM) is associated with disease progression in type 2 diabetes models, and reversal of this modification can restore cardiac function (119). Studies have shown that the occurrence of heart failure may be related to the fragmentation and dysfunction of mitochondria caused by the short-form aggregation of OPA1. The dysfunction of mitochondrial function causes the energy utilization mode of cardiomyocytes to change from lipid to sugar intake (94), which further causes cardiac function insufficiency (See in Figure 5).

The GTPase-dependent mitochondrial fusion proteins MFN1, MFN2, and OPA1 play important roles in maintaining cell homeostasis, cell pressure, calcium regulation, apoptosis, and autophagy. Downregulation or post-translational modification of these fusion proteins have been confirmed as related to a variety of heart diseases (Table 2), however, the specific mechanisms through which MFN1, MFN2, and OPA1 function in cardiomyocytes require further research.

**Table 2:** The role of GTPase-dependent fusion proteins in heart development and heart disease.

**Diseases**	**GTP-ase Dependent dynamin state**	**Methods**	**Effect on the heart**	**References**
Embryogenesis and heart development	MFN(MFN1 and MFN2)	Both MFN1 and MFN2 knocked out	Embryonic lethality	(74)
		Mfn1 and Mfn2 specifically and simultaneously knocked out in cardiac tissue	Dilated cardiomyopathy and Death	(76)
	OPA1	Knocking out *Opa1*	FATAL	(92, 93)
Ischemia-Reperfusion	OPA1	OPA1 expression decreases	Ischemic cardiomyopathy	(95)
		Decreased expression of OPA1	Aggravating ischemia-reperfusion injury	(109)
		Increase or overexpression of OPA1	Maintains heart function	(110)
Cardiac hypertrophy	OPA1	Absence of YME1L s-OPA1 aggravation	Dilated cardiomyopathy and heart failure	(94)
		Hyperacetylation reduces the GTPase activity of OPA1	Hypertrophy of the heart	(107)

## Discussion

The morphology of mitochondria in cardiomyocytes is a highly connected network structure that regulates the key functions of mitochondria through the balance of fission and fusion. Under normal physiological conditions, the fission and fusion of mitochondria are balanced to meet the normal physiological cell functions. In response to cellular stress, mitochondrial fragmentation and excessive fusion have been observed in various diseases, including ischemia-reperfusion injury and heart failure. Under normal physiological conditions, mitochondrial dynamin-related proteins maintain the shape and function of mitochondria to meet the organ needs through their own expression and post-translational modifications. However, under pathological conditions, these fission or fusion proteins can cause mitochondria morphological changes and dysfunction. Post-translational modifications of these proteins, such as acetylation and phosphorylation, can cause excessive fusion or fission of mitochondria; however, this process can be reversed by inhibiting the related proteins.

Therefore, GTP-ase-dependent mitochondrial dynamic-related proteins may represent target protein for the treatment of heart diseases. However, it remains unclear whether it is more beneficial to inhibit mitochondrial fission or promote its fusion to improve heart disease pathology. For instance, although there is a significant body of evidence suggesting that inhibition of DRP1 activity can inhibit mitochondrial fission, while improving heart function and inhibiting the progression of heart disease, a recent study reported that inhibiting mitochondrial fission by enhancing fusion leads to different cardiac outcomes. Meanwhile, up-regulation of MFN2 in the heart can correct excessive mitochondrial fragmentation compared to down-regulation of DRP1. Additionally, the safest strategies will maintain cardiac mitochondrial function (120). Maneechote's team used M1 (2 mg/kg) to enhance mitochondrial fusion to interfere with ischemia-reperfusion in rats and found that the application of M1 before ischemia can reduce infarct size and cardiac apoptosis, exerting the greatest cardioprotective effect. This indicates that myocardial ischemia-reperfusion injury can be reduced by increasing mitochondrial fusion (121). Similarly, the mitochondrial fusion enhancing substance M1 can effectively restore mitochondrial balance and improve diabetic cardiomyopathy in an OPA1-dependent manner (122).

Meanwhile, in different animals and different administration methods, the same type of DRP1 inhibition elicited differing effects. For instance, a recent study showed that injection of Mdivi-1 in advance of myocardial infarction can increase the length of mitochondria in myocardial cells and reduce the area of myocardial infarction in ischemic mice (51). However, another study using pigs as a model for cardiac reperfusion after acute myocardial infarction, reported that injection of Mdivi-1 directly into the coronary artery did not impact mitochondrial morphology nor elicit a protective effect on the heart, which may be related to the dose of Mdivi-1, timing of application, method of administration, etc. (52).Therefore, the timing and methods of intervention in mitochondrial dynamics still need further research.

Collectively, the relevant data has indicated that regulating the fission and fusion balance in mitochondria represents an important strategy for maintaining heart health and function. However, prior to clinical applications for the treatment of heart disease, long-term studies are needed to further investigate the effect of inhibiting mitochondrial motility-related proteins, as well as the appropriate timing and doses for administration.

This review demonstrates that the balance of mitochondrial fission and fusion plays a critical role in maintaining cell and heart function. Meanwhile, the imbalance in these processes likely contributes to the pathophysiological process of heart diseases, thereby providing potential new drug targets for heart diseases. Therefore, mitochondrial fission and fusion balance related proteins warrant further research.

## Author Contributions

BL proposed that mitochondrial-related proteins may play an important role in heart disease based on his previous research and organized the writing of this paper. JL wrote this paper. XS and YY provided guidance, help in the writing process, and the illustrations of the thesis. All authors contributed to the article and approved the submitted version.

## Funding

This study was supported by grants from the Finance Department Project of Jilin Province (No. 2019SCZT004), Education Department Project of Jilin Province (No. JJKH20190055KJ) and Science and Technology Department Project of Jilin Province (No. 2019090502SF).

## Conflict of Interest

The authors declare that the research was conducted in the absence of any commercial or financial relationships that could be construed as a potential conflict of interest.

## Publisher's Note

All claims expressed in this article are solely those of the authors and do not necessarily represent those of their affiliated organizations, or those of the publisher, the editors and the reviewers. Any product that may be evaluated in this article, or claim that may be made by its manufacturer, is not guaranteed or endorsed by the publisher.
